# Does Dapagliflozin influence arterial stiffness and levels of circulating anti-aging hormone soluble Klotho in people with type 2 diabetes and kidney disease? Results of a randomized parallel group clinical trial

**DOI:** 10.3389/fcvm.2022.992327

**Published:** 2022-09-30

**Authors:** Janaka Karalliedde, Nikos Fountoulakis, Dimitra Stathi, Antonella Corcillo, Maria Flaquer, Angeliki Panagiotou, Giuseppe Maltese, Anastasios Mangelis, Salma Ayis, Luigi Gnudi

**Affiliations:** ^1^School of Cardiovascular and Metabolic Medicine and Sciences, King's College London, London, United Kingdom; ^2^School of Population Health and Environmental Sciences, King's College London, London, United Kingdom

**Keywords:** diabetic kidney disease, arterial stiffness, albuminuria, arterial aging, SGLT-2 inhibitors, Klotho

## Abstract

**Objective:**

The mechanisms that explain the cardio-renal benefits of sodium glucose co-transporter 2 (SGLT-2) inhibitors are unknown. The effect of SGLT-2 inhibitors on arterial aging, measured by Aortic Pulse Wave Velocity (Ao-PWV) and Soluble Klotho (s-Klotho), a circulating anti-aging biomarker of arterial health are also unclear.

**Design/Setting:**

A 24-week single center randomized controlled trial (registry number/ EudraCT Number: 2013-004042-42) comparing Dapagliflozin and Ramipril (D+R) versus Ramipril (R) on the primary endpoint of urine albumin excretion rate (AER) and pre-specified secondary endpoints of Ao-PWV and biomarkers of arterial aging [s-Klotho and Fibroblast Growth Factor 23 (FGF-23)]. People with type 2 diabetes who had estimated glomerular filtration rate (eGFR) > 60 ml/min and residual microalbuminuria on maximum tolerated renin angiotensin system (RAS) inhibition were included in this study.

**Results:**

In total, 33 participants (male 73%) were randomized to either D+R (*n* = 17) or R (*n* = 16) arms. After 24 weeks of treatment, Ao-PWV (mean ± SD) did not change significantly from baseline D +R [9.06 ± 1.91 m/s to 9.13 ± 2.03 m/s], and R [9.88 ± 2.12 m/s to 10.0 ± 1.84 m/s]. AER fell significantly by 43.5% (95% CI: −57.36%, −29.56%; *p* < 0.01) in people in the D+ R arm only. We do not observe any significant changes in FGF-23 or s-Klotho. HbA1c and Angiotensin 1–7 fell significantly only in D + R arm.

**Conclusions:**

The combination of Dapagliflozin and Ramipril had no effects on Ao-PWV and s-Klotho which are biomarkers of arterial aging and cardio-renal risk. Our data suggest that the early cardio-renal benefits observed with SGLT-2 inhibitors are unlikely to be related to an improvement in arterial aging.

## Background

Sodium-glucose co-transporter-2 (SGLT-2) inhibitors have demonstrated cardio-renal benefits in large randomized controlled trials in people with diabetic kidney disease (DKD) and residual albuminuria all on renin angiotensin system (RAS) inhibition (1–3). However, the exact mechanisms that may explain these benefits are unclear and several putative mechanisms have been postulated including changes in arterial function and stiffness (4, 5).

Arterial stiffness as measured by aortic pulse wave velocity (Ao-PWV) is an independent predictor of cardiovascular and renal outcomes and a biomarker of arterial aging. We have previously demonstrated a close positive association between Ao-PWV and albuminuria, a biomarker of vascular endothelial dysfunction and independent predictor of cardio-renal outcomes. Adding SGLT-2 inhibitors to RAS blockers reduces albuminuria and prevents cardio-renal disease in people with albuminuric DKD (6, 7).

Soluble Klotho (s-Klotho) and Fibroblast Growth Factor 23 (FGF-23), are involved in the pathogenesis of vascular disease through regulation of endothelial function and vascular calcification (8). SGLT-2 inhibitors have been shown to reduce endothelial inflammation and oxidative stress, which along with the reducing hyperglycemia induced changes in the vasculature and increased vasodilation, can lead to improved vascular function. However, the effect of SGLT-2 inhibitors on Ao-PWV or biomarkers of arterial aging, such as s-Klotho and FGF-23 remains to be elucidated (9).

To further evaluate the role of SGLT-2 Inhibitors on albuminuria, Ao-PWV and other markers of arterial aging, we performed a pre-specified secondary analysis of people with type 2 diabetes (T2DM) with preserved renal function and residual microalbuminuria despite maximum tolerated RAS inhibition who participated in a 24-week randomized controlled trial comparing Dapagliflozin and Ramipril (D+R) vs. Ramipril (R) only.

## Materials and methods

This was a pre-specified secondary analysis of people with T2DM and residual albuminuria despite RAS inhibition who participated in a 24-week prospective randomized trial comparing treatment with D+R to R only.

### Inclusion criteria

Inclusion criteria included age between 35–75 years, residual albuminuria defined as urine albumin creatinine ratio (ACR) > 3 mg/mmol in the preceding 12 months on a maximum tolerated and stable dose of ACE-inhibitor or angiotensin receptor blocker (ARB). All participants needed to have an estimated glomerular function rate (eGFR) more than 60 ml/min. People with history of a CVD event within the past 6 months, on loop diuretics or medications, which could affect sodium balance were excluded. Other exclusion criteria included non-diabetic renal disease, recent or current use of SGLT-2 receptor inhibitor, history of connective tissue disease or inflammatory arthritis, uncontrolled hypertension (systolic blood pressure and diastolic blood pressure >160 and 100 mmHg, respectively), pregnancy and lactation, HbA1c > 12%, recent history of (within 3 years of screening visit) of active malignancy, one or more severe hypoglycaemic episodes within 6 months of screening, New York Heart Association class 3 or 4 cardiac disease and abnormal liver function tests defined as ALT or AST levels > 3 times the upper limit of normal at screening. People with previous hypersensitivity to the active substance, a history of pancreatitis or diabetic ketoacidosis were also excluded.

The trial was conducted in compliance with the principles of the Declaration of Helsinki (1996), and in accordance with all applicable regulatory requirements. Clinical trial registry number / EudraCT Number: 2013-004042-42. The study was approved by Guy's Research Ethics Committee and the Medicines and Healthcare Products Regulatory Agency UK. All participants provided informed consent and were recruited from the outpatient diabetes clinics. The study was funded by a research grant from Astra Zeneca.

### Interventions

Following a 5-week run in phase, participants were randomized by a means of a computer- generated random sequence to either Dapagliflozin 10 mg once daily (OD) and Ramipril 10 mg OD (D+R) or Ramipril 10 mg (R). In the 5-week run in period people who were on RAS inhibition other than ramipril had their ACE inhibitors or ARBs discontinued and replaced by Ramipril.

The primary endpoint of the trial was change in albuminuria as measured by albumin excretion ratio (AER) from timed urine collections from baseline to week 24. Secondary endpoints included Ao-PWV (by applanation tonometry), central aortic blood pressure, components of the RAS (plasma renin activity, aldosterone, ACE-2 and Angiotensin 1-7/1-9 levels) and biomarkers of arterial aging (sKlotho, FGF-23). Other secondary endpoints included changes in Hemoglobin A1c (HbA1c), changes in serum electrolytes (sodium, potassium), urine electrolytes (sodium, potassium, calcium, phosphate, magnesium) and urate, lipids (cholesterol, HDL, LDL, TGL), haematocrit and hemoglobin. All measurements and procedures were performed with the patients in the fasted state and having refrained from nicotine, alcohol, and caffeine for at least the previous 10 h. Brachial blood pressure was measured in triplicate in the supine position by an automated sphygmomanometer (Omron Digital Blood Pressure Monitor HEM 907, Bannockburn, IL). Ao-PWV was determined from carotid and femoral pressure waveforms obtained non-invasively by applanation tonometry (Millar tonometer, Millar Instruments, Houston, TX) using the Sphygmocor system (Atcor, Sydney, Australia) as previously described (6). Central blood pressure determinations including the aortic augmentation index (AIx) were also measured using the same methods.

Urine albumin concentration was measured by immunoturbidimetry using a CobasMiras Plus analyzer (Roche Diagnostics, Rotkreuz, Switzerland) from three timed overnight urine collections, and the median AER was calculated. Serum total cholesterol (enzymatic colorimetry) and creatinine levels were also measured using a Cobas Mira Plus analyzer. Plasmas-Klotho (Immuno-Biological-Laboratories, Hamburg, Germany) and plasma C-terminal FGF-23 (Immunotopics Inc., San Clemente, CA) were measured in duplicate by enzyme-linked immunoassay from samples stored at −80 °C (10–12). Blood samples were immediately centrifuged at 1500 g at 4 °C for 10 min, and the supernatant fractions were stored at −80 °C with no freeze-thaw cycles before analysis. HbA1c was measured by boronate affinity high-performance liquid chromatography (CLC330; Primus, Kansas City, MO). eGFR was calculated using the Chronic Kidney Disease Epidemiology Collaboration Formula. Plasma-sKlotho (Immuno-Biological-Laboratories, Hamburg, Germany) and plasma C-terminal FGF-23 (Immunotopics Inc., San Clemente, CA) were measured in duplicate by enzyme-linked immunoassay from samples as described previously (13). Plasma aldosterone, renin, ACE-2 and angiotensin 1-7/1-9 were measured by enzyme linked immunoassay methods (Cloud-Clone Corp, Houston).

### Statistical analysis

Descriptive statistics were used for the analysis of demographic and clinical features. Data were compared using an unpaired *t* test (for continuous normally distributed variables), Mann–Whitney test (for continuous variables not normally distributed) and χ2 test (for categorical variables). The change in primary and secondary endpoints was analyzed using an analysis of covariance (ANCOVA) with the respective baseline value as covariate. The primary population used in this assessment was the intention to treat population. Endpoint was defined as the last available post-randomization measurement of endpoints. Variables were tested for normality by Shapiro test and Q-Q plots and further on mean, standard deviation and 95% confidence intervals were calculated for the normally distributed variables and median and interquartile range for the non-normally distributed variables. Student's *t*-test for normally distributed variables or Mann-Whitney for non-normally distributed variables were used to compare variables between the two groups. All statistical analysis was done within Rstudio (version 1.3.1073) under R version 4.0.2 (R Foundation for Statistical Computing, Vienna, Austria). A two-tailed *p* value < 0.05 was considered significant.

## Results

In total, 33 participants were randomized (16 in arm R and 17 in arm D+ R). Of the cohort, 72.7% (*n* = 24) were males and 27.3% females (*n* = 9) with T2DM, mean age of 58 years (range 42 to 75 years) and of African-Caribbean 48.5 % (*n* = 16), Caucasian 42.4 % (*n* = 14) and Asian 9.1 % (*n* = 3) ethnicity (Table 1). All participants completed the study except for two people in the R arm, but were both eligible for endpoint analyses. All participants were on metformin, 51.5% was on insulin, 50% on gliclazide, 47% on sitagliptin and 18.8% on Glucagon-like-peptide-1 receptor agonists.

**Table 1:** Baseline data of 33 patients who were eligible for analyses of primary and secondary endpoints randomized to Ramipril or Ramipril + Dapagliflozin treatment arms.

	**Ramipril only (*n* = 16)**	**Ramipril + Dapagliflozin (*n* = 17)**
Gender	Males: 11 (68.8%)	Males: 13 (76.5%)
Mean Age (years)	60 (8.3)	55.5 (9.7)
Mean duration of diabetes (years)	14.2 (4.9)	14.4 (3.9)
Body mass index (kg/m^2^)	30.9 (4.1)	32.9 (3.9)
Systolic blood pressure (mmHg)	138 (21)	139 (14)
Diastolic blood pressure (mmHg)	79 (7)	83 (9)
Aortic pulse wave velocity (m/s)	9.88 (2.12)	9.06 (1.91)
Central systolic blood pressure (mmHg)	128 (17)	120 (15)
Central diastolic blood pressure (mmHg)	81(8)	82 (9)
Central pulse pressure (mmHg)	47 (13)	38 (11)
Augmentation index at HR75 %	19.51 (8.25)	18.85 (6.11)
Albumin excretion rate (μg/min)^*^	108.0 (72.4, 316.0)	35.3 (27.1, 71.5)
eGFR (ml/min/1.73 m^2^)	94.6 (23.5)	98.2 (20.9)
HbA1c (%)	9.2 (1.2)	8.9 (1.1)
Fasting glucose (mmol/l)	9.7 (4.0)	10.8 (3.3)
Serum sodium (mmol/l)	138.5 (3.4)	137.8 (2.7)
Serum potassium (mmol/l)	4.4(0.4)	4.4 (0.5)
Serum creatinine (umol/l)	79.7 (19.5)	74.1(15.4)
Total cholesterol (mmol/l)	4.2 (1.0)	4.1 (1.3)
LDL cholesterol (mmol/l)	2.3 (0.9)	1.9 (1.1)
HDL (mmol/l)	1.3 (0.4)	1.3 (0.3)
Triglycerides (mmol/l)	1.8 (1.7)	2.1 (1.3)
Hemoglobin (g/L)	128.1 (13.9)	140.9 (15.8)
Haematocrit (vol%)	38.7 (3.9)	41.1 (5.1)
ACE-2 (ng/L)	29.01 (45.42)	25.83 (32.41)
Plasma Aldosterone (ng/L)	99.03 (45.36)	84.75 (43.06)
Angiotensin 1.7 (ng/L)	54.29 (22.97)	86.26 (31.74)
Angiotensin 1.9 (ng/L)	166.56 (36.33)	207.55 (91.29)
FGF-23 (ng/L)	12.95 (11.27)	11.67 (13.17)
s-Klotho (ng/L)	851 (433.02)	795.45 (437.94)
Active renin (ng/L)	27.13 (38.46)	48.10 (42.64)

Data mean (standard deviation) or median ^*^interquartile range shown.

eGFR, estimated glomerular filtration rate; SBP, systolic blood pressure; DBP, diastolic blood pressure; FGF-23, Fibroblast growth factor 23; HDL, high density lipoprotein; LDL, low density lipoprotein; ACE, angiotensin converting enzyme.

**Figure 1:**
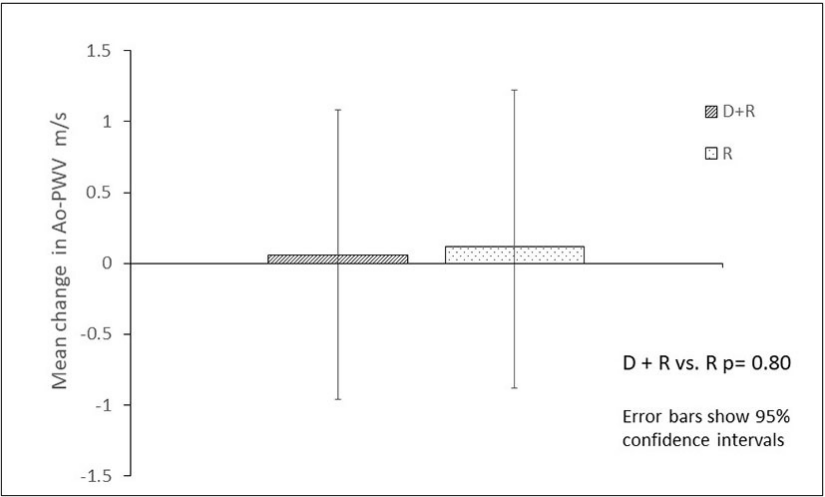
Change in aortic pulse wave velocity in people with type 2 diabetes and kidney disease after 24 weeks treatment with Ramipril only vs. Dapaglifozin + Ramipril.

Table 2, report baseline and end of study values and differences within each arm. Ao-PWV did not change significantly from baseline after 24 weeks in the D +R arm [−0.5 m/s; 95% CI (−1, 2), *p* 0.84] or R arm [0.12 m/s; 95% CI (−0.89, 1.13), *p* 0.81]. Figure 1 summarizes between treatment arm changes in the key secondary endpoint of Ao-PWV at the end of study. There was also no significant change in FGF-23 and s-Klotho in either arm. Over 24 weeks AER fell by 43.5% (95% CI: −57.36%, −29.56%; *p* < 0.01) in people in the D + R arm as opposed to 5% (−48.3%, 38.3%; *p*: 0.36) in the R arm. As expected, mean HbA1c decreased only in D +R treatment arm [mean difference [−1.03% (95% CI: −1.55, −0.51; *p* < 0.001)]. There was however no association between changes in HbA1c and Ao-PWV observed.

**Table 2:** Changes in primary endpoint and selected secondary endpoint variables (baseline to end of study visit) within Ramipril or Ramipril + Dapagliflozin treatment arms.

	**Ramipril only (*n* = 16)**	**Ramipril + Dapagliflozin (*n* = 17)**
**Albumin excretion rate (AER) (ug/min)**		
Baseline, median (IQR)^*^	108.0 (72.4, 316.0)	35.3 (27, 71.1)
End of study, median (IQR)	98.8 (42.3, 184.0)	17.9 (7.8, 119.2)
Difference between visits, median and IQR	−26.1 (−39, 179)	−27.5 (−114, −11)
*p*-value	0.36	<0.001
**Weight (kg)**		
Baseline, mean (SD)	91.5 (13.7)	99.0 (15.1)
End of study, mean (SD)	91.2 (15.1)	97.6 (13.9)
Difference between visits, mean (95% CI)	−0.24 (−1.96, 1.49)	−1.44 (−3.02, 0.14)
*p*-value	0.78	0.07
**Body mass index (kg/m** ^**2**^ **)**		
Baseline, mean (SD)	30.9 (4.1)	32.9 (3.9)
End of study, mean (SD)	30.9 (4.7)	32.6 (3.8)
Difference between visits, mean (95% CI)	0.06 (−0.62, 0.75)	−0.38 (−0.92, 0.17)
*p*-value	0.85	0.16
**Systolic blood pressure (mmHg)**		
Baseline, mean (SD)	138 (21)	139 (14)
End of study, mean (SD)	137 (18)	136 (15)
Difference between visits, mean (95% CI)	−1.65 (−12.52, 9.22)	−2.69 (−13.27, 7.90)
*p*-value	0.75	0.60
**Diastolic blood pressure (mmHg)**		
Baseline, mean (SD)	79 (7)	83 (9)
End of study, mean (SD)	81 (6.)	81 (6)
Difference between visits, mean (95% CI)	1.71 (−3.26, 6.67)	−2.81 (−6.88, 1.25)
*p*-value	0.48	0.16
**Aortic pulse wave velocity (m/s)**		
Baseline, mean (SD)	9.88 (2.12)	9.06 (1.91)
End of study, mean (SD)	10.00 (1.84)	9.13 (2.03)
Difference between visits, mean (95% CI)	0.12 (−0.89, 1.13)	0.06 (−0.96, 1.08)
*p*-value	0.81	0.90
**Central diastolic blood pressure (mmHg)**		
Baseline, mean (SD)	81 (8)	82 (9)
End of study, mean (SD)	82 (6)	80.0 (8)
Difference between visits, mean (95% CI)	0.65 (−4.61, 5.91)	−2.44 (−6.09, 1.22)
*p*-value	0.80	0.18
**Central pulse pressure (mmHg)**		
Baseline, mean (SD)	47 (13)	38 (11)
End of study, mean (SD)	44 (11)	40 (16)
Difference between visits, mean (95% CI)	−2.47 (−8.03, 3.09)	2.31 (−5.11, 9.73)
*p*-value	0.36	0.52
**Augmentation index (%)**		
Baseline, mean (SD)	19.5 (8.3)	18.9 (6.1)
End of study, mean (SD)	20.7 (8.3)	20.9 (8.9)
Difference between visits, mean (95% CI)	1.20 (−2.23, 4.62)	1.07 (−4.01, 6.15)
*p*-value	0.47	0.66
**eGFR (ml/min/1.73 m** ^**2**^ **)**		
Baseline, mean (SD)	94.6 (23.5)	98.2 (20.9)
End of study, mean (SD)	93.4 (20.78)	97.3 (24.0)
Difference between visits, mean (95% CI)	−1.21 (−9.26, 6.83)	−0.96 (−8.79, 6.87)
*p*-value	0.75	0.80
**HbA1c (%)**		
Baseline, mean (SD)	9.2 (1.2)	8.9 (1.1)
End of study, mean (SD)	9.8 (1.7)	7.8 (1.3)
Difference between visits, mean (95% CI)	0.72 (0.08, 1.36)	−1.03 (−1.55, −0.51)
*p*-value	0.03	<0.001
**Fasting glucose (mmol/l)**		
Baseline, mean (SD)	9.7 (4.0)	10.8 (3.3)
End of study, mean (SD)	12.8 (5.3)	9.2 (3.5)
Difference between visits, mean (95% CI)	2.94 (−0.26, 6.13)	−1.34 (−2.93,0.26)
*p*-value	0.07	0.09
**Serum sodium (mmol/l)**		
Baseline, mean (SD)	138.5 (3.4)	137.8 (2.7)
End of study, mean (SD)	136.8 (3.9)	139.8 (2.4)
Difference between visits, mean (95% CI)	−1.76 (−3.18, −0.35)	2.00 (1.09, 2.91)
*p*-value	0.02	<0.001
**Serum potassium (mmol/l)**		
Baseline, mean (SD)	4.4 (0.4)	4.4 (0.5)
End of study, mean (SD)	4.5 (0.4)	4.5 (0.4)
Difference between visits, mean (95% CI)	0.14 (−0.04, 0.31)	0.13 (−0.01, 0.26)
*p*-value	0.12	0.07
**Serum creatinine (umol/l)**		
Baseline, mean (SD)	80.0 (19.5)	74.1 (15.4)
End of study, mean (SD)	79.5 (16.3)	81.9 (29.7)
Difference between visits, mean (95% CI)	−0.12 (−6.90, 6.66)	7.75 (−6.46, 21.96)
*p*-value	0.97	0.26
**Hemoglobin (g/L)**		
Baseline, mean (SD)	128.1 (13.9)	140.9 (15.8)
End of study, mean (SD)	129.2 (18.6)	144.2 (11.9)
Difference between visits, mean (95% CI)	1.18 (−2.65, 5.00)	3.25 (−1.60, 8.10)
*p*-value	0.52	0.17
**Haematocrit (vol%)**		
Baseline, mean (SD)	38.7 (3.9)	41.1 (5.1)
End of study, mean (SD)	39.4 (5.3)	43.2 (3.3)
Difference between visits, mean (95% CI)	0.71 (−0.27, 1.70)	2.08 (−0.06, 4.21)
*p*-value	0.14	0.06
**Angiotensin converting enzyme 2 (ng/L)**		
Baseline, mean (SD)	29.01 (45.42)	25.83 (32.41)
End of study, mean (SD)	38.38 (39.32)	32.81 (39.71)
Difference between visits, mean (95% CI)	7.90 (−14.89, 30.70)	6.98 (−9.87, 23.83)
*p*-value	0.47	0.39
**Aldosterone (ng/L)**		
Baseline, mean (SD)	99.03 (45.36)	84.75 (43.06)
End of study, mean (SD)	116.04 (59.04)	94.48 (41.58)
Difference between visits, mean (95% CI)	16.26 (−13.12, 45.65)	9.73 (−15.54, 35.00)
*p*-value	0.26	0.42
**Angiotensin 1–7 (ng/l)**		
Baseline, mean (SD)	54.29 (22.97)	86.26 (31.74)
End of study, mean (SD)	60.98 (17.30)	76.08 (37.16)
Difference between visits, mean (95% CI)	5.72 (−4.94, 16.38)	−10.17 (−21.34, 0.99)
*p*-value	0.27	0.07
**Angiotensin 1-9 (ng/l)**		
Baseline, mean (SD)	166.56 (36.33)	207.55 (91.29)
End of study, mean (SD)	181.95 (60.94)	182.85 (52.15)
Difference between visits, mean (95% CI)	16.16 (−20.12, 52.43)	−24.71 (−83.50, 34.09)
*p*-value	0.36	0.38
**Active renin (ng/L)**		
Baseline, mean (SD)	27.13 (38.46)	48.10 (42.64)
End of study, mean (SD)	30.91 (40.29)	51.71 (45.78)
Difference between visits, mean (95% CI)	2.54 (−23.23, 28.31)	3.61 (−23.60, 30.82)
*p*-value	0.84	0.78
**s-Klotho (ng/L)**		
Baseline, mean (SD)	851.00 (433.02)	795.45 (437.94)
End of study, mean (SD)	739.37 (337.88)	729.76 (321.96)
Percentage of difference of geometric mean (95% CI)	−34.09 (−38.22,106.41)	−65.69 (−174.2, 42.8)
*p*-value	0.33	0.22
**FGF-23 (pg/ml)**		
Baseline, mean (SD)	12.95 (11.27)	11.67 (13.17)
End of study, mean (SD)	11.78 (15.25)	9.27 (10.71)
Percentage of difference of geometric mean (95% CI)	−1.31 (−3.81, 6.43)	−2.41 (−5, 0.19)
*p*-value	0.59	0.07

Data mean (standard deviation) or median ^*^interquartile range shown.

eGFR, estimated glomerular filtration rate; SBP, systolic blood pressure; DBP, diastolic blood pressure; FGF-23, Fibroblast growth factor 23; HDL, high density lipoprotein; LDL, low density lipoprotein; ACE, angiotensin converting enzyme.

We observed modest changes in serum sodium which increased by 2 mmol/l (95% CI: 1.09, 2.91, *p* < 0.001) and urine magnesium that fell by 2.19 mmol/day (95% CI: −4.32, −0.07; *p* = 0.04) in D +R arm. In contrast no significant changes in components/markers of the RAS were observed (ACE-2, plasma aldosterone and renin activity, Table 2). Angiotensin 1–7 and Angiotensin 1–9 both fell in the D+R arm (−10.17 ng/l; 95% CI: −21.34, 0.99; *p* = 0.07) and (−24.71 ng/l; 95% CI: −83.50, 34.09; *p* = 0.38), respectively, albeit not to a statistically significant level. We also did not observe any significant changes in either group in urinary sodium, phosphate or calcium excretion (from timed urine collections) or serum levels of calcium or phosphate (see Supplementary Table 1).

In our primary and secondary analyses comparing differences between the two arms at the end of the study, 95% CI, *p*-value of ANCOVA test for logged values are shown, we observed significant changes in AER [−0.544 μg/min (95% CI: −1.07, −0.02); *p* = 0.042], HbA1c [−1.7% (95% CI: −2.6, −0.9); *p* < 0.001)], fasting plasma glucose [−1.7 mmol/l (95% CI: −2.6, −0.9); *p* < 0.001] and angiotensin 1–7 [−0.393 ng/l (95% CI: −0.71, −0.07); *p*: 0.017], which were all lower in the D +R arm as compared to the R (Table 3). We also observed that serum sodium and haematocrit were significantly higher at end of study in the D+ R arm compared to the R arm [sodium was higher by 3.6 mmol/l (95% CI: 2, 5.2); *p* < 0.001] and haematocrit 0.05 L/L (95% CI: 0, 0.11; *p* = 0.05), respectively. Overall, dapagliflozin was well tolerated with no serious adverse events (SAE) related to the drug reported (see Supplementary Tables 3, 4 for the list of adverse events).

**Table 3:** Mean difference between Ramipril or Ramipril + Dapagliflozin treatment arms in primary endpoint and selected secondary endpoint variables at end of study.

Albumin excretion rate (AER), mcgg/min	Log mean difference (95% CI): −0.544 (−1.07, −0.02) *p*–value = 0.042
HbA1c %	Mean difference (95% CI): −1.7 (−2.6, −0.9) *p*–value < 0.001
Fasting glucose, mmol/l	Mean difference (95% CI): −3.9 (−7.4, −0.5) *p*–value 0.026
Serum sodium, mmol/l	Mean difference (95% CI): 3.6 (2, 5.2) *p*–value < 0.001
Angiotensin 1,7, ng/l	Log mean difference (95% CI): −0.393 (−0.71, −0.07) *p*–value 0.017
Haematocrit, %	Log mean difference (95% CI): 0.05 (0, 0.11) *p*–value 0.051

## Discussion

In people with T2DM and residual albuminuria despite RAS inhibition, treatment with Dapagliflozin and Ramipril as opposed to Ramipril alone did not significantly change a panel of biomarkers of arterial aging including Ao-PWV, central arterial blood pressure and sKlotho. Consistent with other studies, the addition of dapagliflozin to a RAS inhibitor significantly reduced albuminuria (1–3).

We also observed a significant reduction in HbA1c and fasting glucose levels with dapagliflozin. There are several putative pathophysiological mechanisms that underpin a close relationship between short- and long-term indices glycaemic control and arterial stiffness, including the impact of advanced glycation end-products on arterial wall structure and function (14). However, in our study we did not observe an association between Ao-PWV and changes in fasting glucose or HbA1c glycaemic parameters.

SGLT-2 inhibitors have demonstrated reduction in cardiovascular and renal events in recent trials and several potential mechanisms and pathways have been proposed for these remarkable effects (15, 16). These include potential direct anti-inflammatory protective effects of this class on vascular and renal cells. Indeed, reductions in inflammatory mediators and markers of oxidative stress, have been observed with SGLT-2 inhibition (9, 17, 18).

There is limited and conflicting data on the impact of SGLT-2 inhibitors on arterial aging and stiffness. A study in 16 people with T2DM, who all had blood pressure below 140/90 mmHg and were not on any anti-hypertensive treatment demonstrated an acute treatment effect of dapagliflozin 10 mg OD for 48 h on reducing Ao-PWV (16). Tofoglifolzin treatment for 104 weeks attenuated a rise in brachial ankle pulse wave velocity (baPWV) in people with T2DM (19). However, more than 60% of people in this study were not on RAS inhibition and use of antihypertensive medication was significantly lower in Tofoglifolzin group. A recent study where empagliflozin was used either as monotherapy or in combination with liraglutide demonstrated a reduction in secondary endpoint of Ao-PWV, with empagliflozin use when compared to insulin treatment after 4 and 12 months despite similar HbA1c reduction (20). In this study only 50% of the participants were on RAS inhibitors (20).

In contrast dapagliflozin treatment for 6 weeks did not reduce central arterial pressures or augmentation index as compared to placebo (21). Similarly 12-week treatment of luseogliflozin did not demonstrate any change in cardio-ankle vascular index (CAVI), an index of arterial stiffness (22).

In our study 24 weeks treatment of dapagliflozin added to ramipril did not have any significant impact on Ao-PWV. We and others have previously demonstrated significant reduction in Ao-PWV after 24 weeks of treatment duration with other interventions such as RAS inhibition, which was independent of blood pressure (23, 24). Our findings in this study with dapagliflozin are supported by a recent metanalysis by Wei et al. that included 868 subjects and demonstrated that despite a significant improvement in flow-mediated dilation, there was no significant change in pulse wave velocity with SGLT-2 inhibitors (25).

There are discordant data on the impact of SGLT-2 inhibitors on components of the non-classical RAS pathway that may have putative cardio-renal benefits (26, 27). Treatment with empagliflozin attenuated hyperfiltration in 37 people with type I diabetes not on RAS inhibition and resulted in an increase in urinary levels of angiotensinogen, ACE, ACE-2, rise in plasma angiotensin I, II and renin activity as well as a fall in plasma ACE activity (28, 29). A recent study where empagliflozin was added to RAS inhibitors for 12 weeks in people with CKD with or without diabetes and albuminuria demonstrated a significant increase in angiotensin 1–7, angiotensin I levels and upregulation of plasma renin activity in people with the diabetes (27). In contrast, we did not observe any significant changes in several RAS components, such as ACE-2, aldosterone, angiotensin 1–9 or renin. However, we did observe a fall in circulating angiotensin 1–7 levels. Differences in inclusion criteria, duration of studies and their design as well as differing methodologies used for measurement of RAS components may explain the observed discrepancies (30).

There are no studies reporting the role of SGLT-2 inhibitors in people with diabetes on Klotho, an emerging biomarker of arterial aging and cardio-renal risk. Klotho plays a key role in the arterial aging process by modulating arterial wall inflammation and calcification which may also be influenced by SGLT-2 inhibition (31). Lower levels of sKlotho are an independent predictor of progression of kidney disease (13, 32). An elevated level of FGF-23 is an independent risk factor for cardio-renal disease and increased mortality in people with CKD (33). In our study, treatment with dapagliflozin did not have an impact on either FGF-23 or sKlotho levels. We also did not observe any significant change in urinary calcium or phosphate excretion or changes in serum levels of calcium or phosphate. In contrast to our data in people with stage 3 CKD and albuminuria with and without diabetes 7-day treatment with empagliflozin resulted in a rise on FGF-23 levels only in people with diabetic CKD, but no effect on albuminuria was observed (34). Similarly, data from another study in people with T2DM with albuminuria on RAS inhibition demonstrated that 6 weeks treatment with dapagliflozin increased serum phosphate, plasma PTH, and FGF-23 (35). This effect was independent of concomitant changes in eGFR or urinary albumin excretion (35).

Our study has several limitations and strengths. A major limitation is that we evaluated a small number of participants. Our study was based at one center and had a SGLT-2 inhibitor treatment duration of 24 weeks. Much larger and longer duration trials are needed to confirm our results. Strengths of our study include the very specific inclusion criteria of people with type 2 diabetes and early stages of chronic kidney disease (stage 1 and 2) with residual albuminuria despite RAS inhibition. In contrast to other studies, we standardized the RAS inhibition used with all people being on ramipril during trial. Furthermore, we evaluated arterial stiffness using the gold standard measure of Ao-PWV. The 24-week treatment period with dapagliflozin is longer than many other similar proof of concept trials and indeed changes in Ao-PWV and sKlotho have been observed with other interventions of shorter duration (10, 23).

## Conclusions

We have demonstrated that the combination of dapagliflozin and ramipril treatment does not influence Ao-PWV, measures of central arterial stiffness or sKlotho as compared to ramipril alone in people with type 2 diabetes and residual microalbuminuria despite optimal RAS blockade. Our results suggest that the observed early cardio-renal benefits of SGLT-2 inhibitors are unlikely to be related to improvements in arterial stiffness or arterial aging.

## Data availability statement

Requests for access to data should be addressed to the corresponding author.

## Ethics statement

The study was approved by Guy's Research Ethics Committee and the Medicines and Healthcare Products Regulatory Agency UK. The patients/participants provided their written informed consent to participate in this study.

## Author contributions

JK and LG designed the research study, interpreted the data, and drafted the article. NF, DS, MF, GM, AP, and AC collected and interpreted the data and contributed to the manuscript. AM and SA contributed and led on data analysis and interpretation. All authors have reviewed the article and approved the final draft.

## Funding

This work was funded by a research grant from Astra Zeneca. The funder was not involved in the study design, collection, analysis, interpretation of data, the writing of this article or the decision to submit it for publication.

## Conflict of interest

The authors declare that the research was conducted in the absence of any commercial or financial relationships that could be construed as a potential conflict of interest.

## Publisher's note

All claims expressed in this article are solely those of the authors and do not necessarily represent those of their affiliated organizations, or those of the publisher, the editors and the reviewers. Any product that may be evaluated in this article, or claim that may be made by its manufacturer, is not guaranteed or endorsed by the publisher.
