# Supplementary material for: Does Dapagliflozin influence arterial stiffness and levels of circulating anti-aging hormone soluble Klotho in people with type 2 diabetes and kidney disease? Results of a randomized parallel group clinical trial
Source: Front Cardiovasc Med. 2022 Sep 30;9:992327. doi: 10.3389/fcvm.2022.992327 (PMC9562264; doi:10.3389/fcvm.2022.992327)
Supplement: Supplementary file 1 [file Data_Sheet_1.docx]

**Supplemental Table 1: Changes in serum calcium, phosphate and urine electrolytes in Ramipril only and Ramipril and Dapagliflozin treatment arms**

|  | **Ramipril only**  **(n=15)** | **Ramipril and Dapagliflozin (n=16)** |
| --- | --- | --- |
| Serum Calcium (mmol/l)  Baseline, mean (SD)  End of study, mean (SD)  Difference between visits, mean (95% CI), p-value | 2.4 (0.08)  2.4 (0.1)  -0.01 (-0.11, 0.08)  0.7 | 2.4 (0.07)  2.4 (0.09)  0.01 (-0.05, 0.08)  0.6 |
| Serum Phosphate (mmol/l)  Baseline, mean (SD)  End of study, mean (SD)  Difference between visits, mean (95% CI), p-value | 1.1 (0.8)  1.06 (0.11)  -0.04 (-0.18, 0.1)  0.4 | 1.08 (0.2)  1,10 (0,1)  0.1 (-0.13, 0.33)  0.3 |
| Urinary sodium (mmol/day)  Baseline, mean (SD)  End of study, mean (SD)  Difference between visits, mean (95% CI), p-value | 220.36 (167.86)  241.25 (209.61)  20.89 (-106.98, 148.76)  0.73 | 217.89 (139.28)  187.71 (177.01)  -31.99 (-108.70, 44.72)  0.39 |
| Urinary potassium (mmol/day)  Baseline, mean (SD)  End of study, mean (SD)  Difference between visits, mean (95% CI), p-value | 89.65 (72.06)  85.58 (65.12)  -4.07 (-47.67, 39.53)  0.85 | 88.62 (54.79)  71.79 (37.77)  -17.81 (-47.54, 11.91)  0.22 |
| Urinary calcium (mmol/day)  Baseline, mean (SD)  End of study, mean (SD)  Difference between visits, mean (95% CI), p-value | 4.98 (4.47)  4.35 (4.18)  -0.62 (-3.13, 1.88)  0.60 | 5.99 (4.95)  5.81 (6.58)  0.09 (-1.73, 1.90)  0.92 |
| Urinary phosphate (mmol/day)  Baseline, mean (SD)  End of study, mean (SD)  Difference between visits, mean (95% CI), p-value | 23.50 (13.22)  19.51 (10.29)  -3.99 (-10.59, 2.60)  0.22 | 33.20 (22.78)  29.38 (21.06)  -4.03 (-16.16, 8.09)  0.49 |
| Urinary magnesium (mmol/day)  Baseline, mean (SD)  End of study, mean (SD)  Difference between visits, mean (95% CI), p value | 6.34 (5.08)  5.63 (4.76)  -0.71 (-3.20, 1.78)  0.55 | 7.71 (4.82)  5.91 (3.33)  -2.19 (-4.32, -0.07)  0.04 |
| Urinary urate (mmol/day)  Baseline, mean (SD)  End of study, mean (SD)  Difference between visits, mean (95% CI), p-value | 4.21 (3.05)  3.79 (2.57)  -0.42 (-2.26, 1.42)  0.63 | 4.19 (1.90)  4.18 (2.74)  -0.15 (-1.17, 1.48)  0.81 |

**Supplemental table 2: Summary of adverse events**

| **Event** | **Dapagliflozin and Ramipril** | **Ramipril** |
| --- | --- | --- |
| Non-serious AEs | 88.23% (15/17) | 68.75% (11/16) |
| SAEs | 23.5% (4/17) | 6.25% (1/16) |
| Discontinuation of IMP due AEs/SAEs | 0% (0/17) | 0% (0/16) |
| Deaths | 0% (0/17) | 0% (0/16) |

**Supplemental Table 3: Adverse events**

| **Arm** | **Event** | **System organ class** | **Causality related to intervention** |
| --- | --- | --- | --- |
| Ramipril + Dapagliflozin | Weakness | Musculoskeletal disorders | Unlikely |
|  | Peptic ulcer disease | Gastrointestinal disorders | Unlikely |
|  | Candidiasis at lower oesophagus | Infectious diseases | Unlikely |
|  | Angina | Cardiovascular disorders | Unlikely |
|  | Diarrhoea | Gastrointestinal disorders | Unlikely |
|  | Hypokalaemia | Electrolyte disorders | Unlikely |
|  | Right upper quadrant pain | Gastrointestinal disorders | Unlikely |
|  | Constipation | Gastrointestinal disorders | Possibly |
|  | Fungal genital infection | Urogenital disorders | Possibly |
|  | Atrial fibrillation | Cardiovascular disorders | Unlikely |
|  | Dry mouth | Oral mucosal diseases | Likely |
|  | Back pain | Musculoskeletal disorders | Possibly |
|  | Urinary frequency exacerbation | Urinary disorders | Possibly |
|  | Cough | Respiratory disorders | Possibly |
| Ramipril | Cough | Respiratory disorders | Possibly |
|  | Bilateral knee pain | Musculoskeletal disorders | Unlikely |
|  | Hypoglycaemia | Endocrine disorders | Unlikely |
|  | Foul smelling urine | Urinary disorders | Unlikely |
|  | Pain in the legs | Musculoskeletal disorders | Unlikely |
|  | Urinary tract infection | Urinary disorders | Unlikely |
|  | Flu-like symptoms | Infectious diseases | Unlikely |
|  | Macroscopic haematuria | Urinary disorders | Unlikely |
|  | Gastritis | Gastrointestinal disorders | Unlikely |
|  | Palpitations | Cardiovascular disorders | Unlikely |
|  | Fall | Mechanical fall | Unlikely |

**Supplemental table 4: Serious adverse events**

| **Arm** | **Event** | **System organ class** | **Causality related to intervention** |
| --- | --- | --- | --- |
| Ramipril + Dapagliflozin | Resection of endometrial fibroids | Gynaecology disorders | Unlikely |
|  | Endophtalmitis after an eye injection | Eye disorders | Unlikely |
|  | Right Orbital Inflammatory Syndrome (orbital infection) | Eye disorders | Unlikely |
|  | Hyperglycaemia with dehydration and paroxysmal atrial fibrillation | Endocrine disorders/Cardiovascular disorders | Unlikely |
| Ramipril | Hypersensitive airways disease on background of undiagnosed chronic airways disease | Respiratory disorders | Unlikely |
